# Efficacy of the induced pluripotent stem cell derived and engineered CD276-targeted CAR-NK cells against human esophageal squamous cell carcinoma

**DOI:** 10.3389/fimmu.2024.1337489

**Published:** 2024-03-19

**Authors:** Xiaolan Lin, Tian Guan, Yien Xu, Yun Li, Yanchun Lin, Shaobin Chen, Yuping Chen, Xiaolong Wei, Dongsheng Li, Yukun Cui, Yan Lin, Pingnan Sun, Jianmin Guo, Congzhu Li, Jiang Gu, Wei Yang, Haoyu Zeng, Changchun Ma

**Affiliations:** ^1^ Department of Radiation Oncology, Cancer Hospital of Shantou University Medical College, Shantou, Guangdong, China; ^2^ Guangdong Procapzoom Bioscience Inc, Guangzhou, Guangdong, China; ^3^ Procapzoom-Shantou University Medical College iPS Cell Research Center, Shantou, Guangdong, China; ^4^ Department of Thoracic Oncology, Cancer Hospital of Shantou University Medical College, Shantou, Guangdong, China; ^5^ Department of Pathology, Cancer Hospital of Shantou University Medical College, Shantou, Guangdong, China; ^6^ Guangdong Provincial Key Laboratory for Breast Cancer Diagnosis and Treatment, Cancer Hospital of Shantou University Medical College, Shantou, Guangdong, China; ^7^ Department of Medical Imaging, the Second Affiliated Hospital, Shantou University Medical College, Shantou, Guangdong, China; ^8^ Department of Stem Cell Research Center, Shantou University Medical College, Shantou, Guangdong, China; ^9^ Division of Life Science and State Key Lab of Molecular Neuroscience, Hong Kong University of Science and Technology, Hong Kong, Hong Kong SAR, China; ^10^ Department of Gynecological Oncology, Cancer Hospital of Shantou University Medical College, Shantou, Guangdong, China; ^11^ Guangdong Provincial Key Laboratory of Infectious Diseases and Molecular Immunopathology, Shantou University Medical College, Shantou, Guangdong, China; ^12^ Guangzhou Bay Area Institute of Biomedicine, Guangdong Lewwin Pharmaceutical Research Institute Co., Ltd., Guangdong Provincial Key Laboratory of Drug Non-Clinical Evaluation and Research, Guangdong, China; ^13^ Key Laboratory of Molecular Target & Clinical Pharmacology and State Key Laboratory of Respiratory Disease, School of Pharmaceutical Sciences, Guangzhou Medical University, Guangzhou, Guangdong, China

**Keywords:** iPSC CD276-targeted CAR-NK, patient specific organoid, normal control PSO, pre-clinical models, esophageal squamous cell carcinoma

## Abstract

**Introduction:**

Chimeric antigen receptor natural killer (CAR-NK) cells have been found to be successful in treating hematologic malignancies and present potential for usage in solid tumors.

**Methods:**

In this study, we created CD276-targeted CAR-expressing NK cells from pluripotent stem cells (iPSC CD276-targeted CAR-NK cells) and evaluated their cytotoxicity against esophageal squamous cell carcinoma (ESCC) using patient-specific organoid (PSO) models comprising of both CD276-positive and CD276-negative adjacent epithelium PSO models (normal control PSO, NC PSO) as well as primary culture of ESCC cell models. In addition, *in vitro* and *in vivo* models such as KYSE-150 were also examined. iPSC NK cells and NK-free media were used as the CAR-free and NK-free controls, respectively.

**Results:**

The positive CD276 staining was specifically detected on the ESCC membrane in 51.43% (54/105) of the patients of all stages, and in 51.35% (38/74) of stages III and IV. The iPS CD276-targeted CAR-NK cells, comparing with the iPS NK cells and the NK-free medium, exhibited specific and significant cytotoxic activity against CD276-positive ESCC PSO rather than CD276-negative NC PSO, and exhibited significant cytotoxicity against CD276-expressing cultured ESCC cells, as well as against CD276-expressing KYSE-150 *in vitro* and in BNDG mouse xenograft.

**Discussion:**

The efficacy of the iPSC CD276-targeted CAR-NK cells demonstrated by their successful treatment of CD276-expressing ESCC in a multitude of pre-clinical models implied that they hold tremendous therapeutic potential for treating patients with CD276-expressing ESCC.

## Introduction

Adoptive cell therapy (ACT) has the potential for cancer treatment using chimeric antigen receptor (CAR) to redirect immune cells’ specificity against cancer-specific antigens. Natural killer (NK) cells that express CARs (CAR-NK cells) have been considered the possible successor of cellular therapy after the revolutionary success of CAR-T cells (1–3). In the recent past, clinical trials have demonstrated the efficacy of CAR-NK cell therapy in treating patients with CD19-expressing hematologic malignancies with minimal toxicity. Moreover, CAR-NK cell therapy is predicted to provide therapeutic benefits for patients suffering from solid tumors (4, 5).

The use of CAR-T cells has transformed traditional cancer treatment, however, their clinical application is restricted by the toxicity and potential complications associated with their manufacturing, transplant, and allogeneic transplantation, resulting in a risk of cytokine release syndrome, neurotoxicity, logistical complexity, cost, and time-to-treatment (2, 6, 7). Also, the treatment of solid tumors with CAR-T cells is challenging because of the immunosuppression of the tumor microenvironment (7, 8).

Due to their broad availability and safety profile, NK cells have attained attention as an integral source of cells for CAR-based immune cell therapies. As a component of the innate immune system, NK cells provide the first line of defense against infections and malignant cells. They do not require antigen sensitization to produce cytokines and mediate cytotoxicity. Additionally, NK cells can communicate and activate other immune cells (1, 9). The generation of NK cells for immunotherapy is achievable from several sources, including expanded autologous, allogeneic peripheral blood, umbilical cord blood, hematopoietic stem cells, induced pluripotent stem cells, and NK-92 cell line. Consequently, CAR-NK cells have developed a critical role in the forthcoming cellular therapies against cancer. Numerous preclinical and clinical studies have been conducted to enhance engineered innate immune cell’s antitumor activity, and CAR-NK cells derived from different sources and strategies have been developed (1, 2, 9). Recently, allogeneic umbilical cord blood-derived anti-CD19 CAR-NK cell therapy accomplished curing effects in patients with relapsed/refractory non-Hodgkin’s lymphoma or chronic lymphocytic leukemia demonstrated through a phase 1/2 clinical trial, without graft-versus-host disease, cytokine release syndrome, or neurological events (4). The efficacy of CAR-NK cell therapy in hematologic malignancies highlights its potential for use in other cancer types.

Chimeric antigen receptor T cell CAR-T therapy has demonstrated promising advances in clinical immunotherapy. However, its effectiveness against most solid tumors is constrained by several obstacles, including limited tumor trafficking and infiltration, the presence of an immunosuppressive tumor microenvironment, as well as potential adverse events associated with CAR-T therapy. To address these challenges, CAR-Natural Killer CAR-NK cells have emerged as a complementary or alternative approach for treating solid tumors. CAR-NK cells offer several advantages over CAR-T cells, such as their non-MHC-restricted recognition capacity and reduced toxicity. They possess innate abilities to recognize non-self-cells, engage in direct and indirect killing mechanisms through CAR and ADCC, respectively, exhibit self-recognition of normal cells through KIR, and pose a reduced risk of cytokine release syndrome (CRS), immune effector cell-associated neurotoxicity syndrome (ICANS), and graft-versus-host disease (GvHD). Moreover, CAR-NK cells can be generated on a larger scale from diverse sources, making them promising off-the-shelf products (10, 11). Growing evidence of the efficacy of CAR-NK cells in patient-derived organoid-based pre-clinical models highlights their potential as a clinical therapeutic option for treating solid tumors (12–15). However, CAR-NK cells may encounter challenges such as limited tumor infiltration, restrictions in CAR transduction efficiency, limited survival, and persistence in the immunosuppressive tumor microenvironment.

Esophageal cancer (EC) is a significant global health issue, ranking as the ninth most common cancer and the sixth leading cause of cancer-related deaths worldwide (16, 17). This malignancy comprises two primary histological subtypes with distinct epidemiological and clinical characteristics: esophageal squamous cell carcinoma (ESCC) and esophageal adenocarcinoma (EAC). ESCC accounts for approximately 70% of all EC cases, and nearly half of the 500,000 new ESCC cases reported annually worldwide occur in China (17, 18). ESCC carries a poor prognosis due to its late-stage diagnosis, high recurrence, and mortality rates, despite advances in conventional treatments such as surgery, radiotherapy, and chemotherapy (19–21), and immune checkpoint inhibitors targeting the programmed death 1 (PD-1)/programmed death-ligand 1 (PD-L1) pathway (22–25). The current targeted therapies for ESCC are limited, mainly due to a lack of specific diagnostic and therapeutic biomarkers.

CD276, also known as B7-H3, is a type I transmembrane glycoprotein that overexpresses extensively in a variety of solid tumors, including breast cancer, esophageal squamous cell carcinoma, head and neck squamous cell carcinoma, lung cancer, and prostate cancer, and is termed as a pan-solid tumor-related cell surface antigen. CD276 has relatively limited expression in non-immune resting fibroblasts, endothelial cells, osteoblasts, amniotic fluid stem cells, and induced immune cells (26–28). CD276 has been linked with poor prognosis, aggressive clinical characteristics, and low tumor-infiltrating lymphocytes (29, 30). A study of melanoma spheroids revealed that CD276-targeted CAR-NK-92 cells can invade tumors efficiently and maintain cytotoxic activity in an immunosuppressive environment (31). Recently, both CD276-targeted CAR-T and CD276-targeted CAR-NK92 cells have been studied in preclinical models, demonstrating favorable therapeutic response in CD276-expressing malignancies (28, 31–34). However, there is no available information about the usage of CAR-NK cells in treating esophageal squamous cell carcinoma (ESCC).

Due to the accessibility and specificity of CD276-targeted CAR in former studies (3, 35, 36), we developed the iPSC CD276-targeted CAR-NK cells and appraised their cytotoxicity against CD276-expressing ESCC patient-specific organoids and primary cultured ESCC cells, along with CD276-expressing human ESCC cell line (KYSE-150) *in vitro*, and in BNDG mouse models. The considerable effectiveness of the iPSC CD276-targeted CAR-NK cells against human ESCC demonstrated in the preclinical trial underscores the need for further clinical research.

## Materials and methods

### Key resources table

**Table:** Key reagent and resources

REAGENT or RESOURCE	SOURCE	IDENTIFIER
Antibodies		
goat anti-human CD276 antibody	R&D Systems	Cat# AF1027, RRID: AB_354546
rabbit anti-human CD276 antibody	Abcam	Cat# ab227670
rabbit anti-CD56 polyclonal antibody	Proteintech	Cat# 14255-1-AP, RRID: AB_2149421
rabbit anti-CD16 polyclonal antibody	Proteintech	Cat# 16559-1-AP, RRID: AB_2878279
Polymer detection systems for immune-histological staining	Zhongshan Golden Bridge Biology	Cat# PV-9003, RRID: AB_2814979
Polymer detection systems for immune-histological staining	Zhongshan Golden Bridge Biology	Cat# PV-9000
peroxidase-conjugated secondary antibodies	Abcam	Cat# ab205718, RRID: AB_2819160
GAPDH	Ambion	Cat# AM4300, RRID: AB_2536381
Cell lines		
KYSE-140	–	Cat# Cancer cell line, RRID: CVCL_1347
KYSE-150	–	Cat# Cancer cell line, RRID: CVCL_1348
Critical commercial assays		
Bicinchoninic Acid protein assay kit	Sangon Biotechnology	Cat# C503021
STEMdiff™ NK Cell Kit	STEMCELL	Cat# 100-0170
Cell Counting Kit-8	Beyotime Biotechnology	Cat# C0038
Animals		
NOD -Prkdcscid IL2rgtm1/Bcgen mice	Biocytogen Pharmaceuticals	Cat# 110586
Software and algorithms		
GraphPad Prism v.8	GraphPad Software	RRID: SCR_002798
IBM SPSS Statistics 25 software	IBM SPSS Software	RRID: SCR_019096
Other		
automated chemiluminescence image analysis system Tanon 5200	Tanon	Model: Tanon 5200 Multi
xCELLigence Real-Time Cell Analysis system	Agilent	Model: xCELLigence RTCA S16
enzyme-labeled instrument	Potenov Inc.	Cat# PT-3502B

### Resource availability

#### Materials availability

The associated patents of iPSC CD276-targeted CAR-NK cells and patient specific organoid are pending. There are restrictions to the availability of iPSC CD276-targeted CAR-NK cells (human resources) and patient specific organoid (clinical resources) due to the clinical ethics. iPSC CD276-targeted CAR-NK cells and patient specific organoid generated in this study will be made available on request, but we may require a payment and/or a completed materials transfer agreement if there is potential for commercial application.

### Experimental model and subject details

#### PSO model protocol for immunotherapy of CD276-targeted CAR-NK cells against ESCC

The living PSOs were cultured in 96 well plate with 100 µL PSO culture medium (Guangdong Procapzoom Biosciences, Guangdong, China) for each well. For treatment, NK cells or CAR-NK cells were suspended in NK cells culture medium at the density of 1 × 10^5^ cells/ml and added into 96 well plate for 100 µL/well, the control groups were added with 100 µL NK cells culture medium into each well. After treatment, the PSO and NK/CAR-NK cells were co-cultured in the 96 well plate in 5% CO2 incubator at 37°C for 6 days and monitored periodically.

#### The protocol for patient-specific primary cells of ESCC co-cultured with iPSC-derived CD276-targeted CAR-NK cells detected by CCK8 assay

The viability of ESCC cells co-cultured with the iPSC CD276-targeted CAR-NK, iPSC NK and the blank NK-culturing medium were analyzed by Cell Counting Kit-8 (CCK8) in a dose-dependent manner according to the manufacturer’s protocols. Briefly, ESCC cells were resuspended at a density of 2 × 10^5^ cells/ml, and seeded into 96-well plate, with 100 μL in each well for 24 hours at 37°C. Followed by treated with different concentrations (0.5 and 1×10^5^ cells/ml) of the iPSC CD276-targeted CAR-NK cells and the iPSC NK cells at a same dose respectively, and incubated for 12 hours, the NK-culturing medium and NK cells suspension were removed, washed and replaced by new medium. 10 µL CCK8 regent was added to each well, the plates were shielded from light and incubated for 2 ~ 4 hours at 37°C. Optical density (OD) values were measured by enzyme-labeled instrument at a wavelength of 450 nm using ESCC cells without co-culturing with NK cells as blank controls.

#### The protocol for ESCC cell line co-cultured with iPSC-derived CD276-targeted CAR-NK cells detected by xCELLigence Real-Time Cell Analysis system

The cytotoxicity of CD276-CAR-NK to human ESCC line (KYSE150) was measured by the xCELLigence Real-Time Cell Analysis system. RPMI with 10% FBS was added to E-Plate 16 (Agilent) at 50 μL per well for measuring background impedance. Add 100 μL of KYSE150 cell suspension to each well of E-Plate 16 at a density of 1 × 10^5^ cells/ml. Place the 16-well plate at 37°C and 5% CO2, measure impedance every 15 minutes for approximately 22 h. After that, half of the RPMI in the 16-well of E-Plate was replaced with NK-culturing medium, which was used as a blank control group. The experimental group used 50 μL iPSC-NK or CD276-CAR-NK cell suspension diluted to a density of 1 × 10^5^ cells/mL. Impedance is measured every 15 minutes for approximately 24 hours. The cell index of each group measured by xCELLigence RTCA was normalized across the timeline.

#### Humanized mouse model protocol for iPSC-derived CD276-targeted CAR-NK cell-based immunotherapy of ESCC

6-week-old, 20-grams-weight female B-NDG (NOD -Prkdcscid IL2rgtm1/Bcgen) mice obtained from Biocytogen Pharmaceuticals (Beijing) Co., Ltd (Jiangsu, China) were raised in the specific pathogen-free (SPF) breeding unit of the Experimental Animal Center of SUMC. Each mouse was injected with 100 μL of PBS containing 4 × 10^6^ Kyse150 cells subcutaneously in the right flank. The ESCC cell line xenografts were randomized divided into 3 groups when the subcutaneous tumor volume of the mice reached 100 mm^3^ according to body weight at baseline, each group contains 5 mice. 100μL of PBS suspension containing 1×10^6^ CD276 CAR-NK cells and iPSC-NK cells were injected through the tail vein of the mouse. The control group was also injected with 100μL normal saline, which was repeated once a week for 3 consecutive weeks. We used an electronic vernier caliper to measure and record the maximum longitudinal diameter of the tumor as the length and the maximum transverse diameter perpendicular to the length as the width, then calculate the tumor volume by the formula: tumor size (mm^3^) = ½ (length × width ^2^) each day. All mice were euthanized after one month. The subcutaneous tumor, liver, spleen and lung were fixed with paraformaldehyde, embedded in paraffin, and then sectioned for immunohistochemical staining.

#### Study approval

The study was approved by the Ethical Committee of the Cancer Hospital of Shantou University Medical College (No. 2022103) and the informed consent was obtained from all participants. All animal experiments have been approved by the Medical Animal Care & Welfare Committee of Shantou University Medical College (No. SUMC2022-293).

### Method details

#### Human ESCC samples and ESCC Cell lines

Formalin-fixed, paraffin-embedded ESCC and adjacent normal esophagus tissues 2cm away from the proximal edge of the ESCC were collected from 105 patients in the archives of the Cancer Hospital of Shantou University Medical College (SUMC), from 2012 to 2017. A total of 25 sets of fresh ESCC and the adjacent normal esophagus epithelium tissues were collected and processed immediately after surgery in the Cancer Hospital between February and August in 2021. Clinical data from these 105 ESCC patients were obtained, and the characteristics of these patients are detailed in **Table 1**. The two ESCC cell lines (KYSE-140 and KYSE-150) were kindly donated by Prof. Liyan Xu (the Guangdong Provincial Key Laboratory of Infectious Diseases and Molecular Immunopathology and the Oncological Pathology Laboratory of Shantou University Medical College).

**Table 1:** Correlation of ESCC CD276 expression to clinical parameters.

Clinical Parameters		Cases	CD276 expression		χ^2^	P-vaule
			+(%)	-(%)		
Gender	Male	80	39(48.8)	41(51.2)	0.965	0.326
	Female	25	15(60.0)	10(40.0)		
Age	<60	49	25(51)	24(49)	0.006	0.938
	≥60	56	29(51.8)	27(48.2)		
Depth of invasion (T)	T1+T2	13	4(30.8)	9(69.2)	2.535	0.111
	T3+T4	92	50(54.3)	42(45.7)		
Nodal metastasis(N)	N0	44	26(59.1)	18(40.9)	1.780	0.182
	N1,2,3	61	28(45.9)	33(54.1)		
TNM stage	I+II	31	16(51.6)	15(48.4)	0.001	0.980
	III+IV	74	(51.4)	36(48.6)		
Tumor site	Upper	17	7(41.2)	10(58.8)	6.094	**0.047**
	Middle	65	30(46.2)	35(53.8)		
	Lower	23	17(73.9)	6(26.1)		
Differentiation	Well	42	22(52.4)	20(47.6)	2.567	0.277
	moderately	45	20(44.4)	25(55.6)		
	poorly	18	12(66.7)	6(33.3)		

The results in bold are statistically significant.

#### Immunohistochemistry

The 4% paraformaldehyde-fixed, paraffin-embedded ESCC and adjacent esophagus tissues were cut into 4μm-thick sections. They were deparaffinized and rehydrated in graded concentrations of ethanol to distilled water. Sections were placed into 10 mM citrate buffer (pH 6.0), heated to boiling in pressure cooker for 20 min, and then cooled to room temperature. After rinsing with PBS, the sections were inserted in 3% hydrogen peroxide (H_2_O_2_) for 30 min and rinsed again in 0.01 M PBS at room temperature. After blocking with 10% normal horse serum for 1 hour, the primary antibodies, goat anti-human CD276 antibody (1:100) or rabbit anti-human CD276 antibody (1:100), rabbit anti-CD56 polyclonal antibody (1:100) or rabbit anti-CD16 polyclonal antibody (1:100) were added to the corresponding sections respectively, and incubated overnight at 4°C. Polymer detection systems for immune-histological staining were used, which gives a red color. Sections were then counterstained with hematoxylin. Primary antibody was replaced by PBS as a negative control.

#### Western blot

Cells after culture were washed twice with DPBS at 4°C and then lysed with Radio Immunoprecipitation Assay (RIPA) lysis buffer. Human tumor tissues were pestled in RIPA lysis buffer. The protein concentration of each sample was detected with Bicinchoninic Acid (BCA) protein assay kit. Equal amount of sample protein was size fractioned by 12% polyacrylamide gel electrophoresis (PAGE), and transferred to a polyvinylidene fluoride (PVDF) membrane. Non-specific binding was blocked by incubating in TBST 5% BSA plus 1% Triton X-100 solution for 1 hour. Followed by incubation with rabbit anti-human CD276 antibody (1:400) overnight at 4°C, species specific peroxidase-conjugated secondary antibodies (1:10,000) were applied to membrane for 1 hour at room temperature. Bands were detected using an automated chemiluminescence image analysis system Tanon 5200 (Tanon, Shanghai, China). All loading samples were normalized by staining with GAPDH (1:300).

#### CD276-CAR-modified induced pluripotent stem cells construction and NK cell differentiation

The CD276-CAR consists of the extracellular domain containing the CD276 scFv sequence, the transmembrane region, the intracellular domain 4-1BB and the CD3ζ signal peptide. The corresponding sequences were cloned into the pWXLD lentiviral vector, embedded in lentivirus and transfected into HEK 293T cells. The procedure was performed in a medium consisting of 1 µg/ml pMD.2G, 2 µg/ml psPAX2, 10 µg/ml PEI and Opti-MEM. About 6 hours after transfection, the medium was changed to high-glucose DMEM, and the supernatant was collected every 24 hours. The lentivirus was concentrated from the supernatant with virus concentration reagent (Accurate Biology Inc., Hunan, China. Cat# AG51001). For iPSC cell transduction, each well of the 12-well plate was added with 1-3×106 iPSC cells, concentrated viral particles, and 8 µg/ml polybrene. After 24 hours, the transfected iPSC cells were returned to the original medium. The procedure of inducing CAR-iPSC/iPSC cells to CAR-NK/iPSC-NK cells differentiation was following the steps of STEMdiff™ NK Cell Kit. Briefly, CD34+ hematopoietic progenitor cells were generated from iPSCs using STEMdiff™ NK Cell Kit firstly. iPSCs were seeded in AggreWell™400 6-well plate (STEMCELL, Catalog # 34421) and cultured with EB Formation Medium. At day 2, the medium was changed to EB Medium A, and at day 3, transferred to EB Medium B, then the CD34+ hematopoietic progenitor cells could be harvested at day 12. Secondly, the isolated CD34+ hematopoietic progenitor cells were seeded in coated AggreWell™400 24-well plate (STEMCELL, Catalog # 34411) and cultured with StemSpan™ Lymphoid Progenitor Expansion Medium for approximately two weeks. At day 14, the medium was changed to StemSpan™ NK Cell Differentiation Medium and culturing for two weeks, then CAR-NK/iPSC-NK cells could be harvested at day 28.

In order to validate the CD276-CAR-NK model prior to the preclinical experiment, we performed flow cytometry analysis to examine the phenotype of the cells, starting from iPSCs expressing CD276-CAR, Oct4, SSEA3, SSEA4, TRA-1-60, TRA-1-81 and Nanog. Further differentiation led to the emergence of CD276-CAR-NK progenitors (CD276-CAR+, CD45+, CD34+), finally producing mature CD276-CAR-NK cells (CD276-CAR+, CD45+, CD3-, CD56+, CD16+) (**Supplementary Figure 3**).

#### Generation of PSOs and patient-specific primary cells

The manufacture of PSO and patient-derived primary cells was partially modified following the production procedure of Driehuis, E. et al. (37). Tumor tissues and adjacent normal tissues were surgically obtained from ESCC patients and kept viable in storage buffer prepared by Guangdong Procapzoom Biosciences, Inc., (Guangdong, China). The tissue was then transferred to a cell culture chamber. After washing 3-6 times with washing buffer, the tissue was cut into pieces under 200 μm diameter in storage buffer and filtered through a 100 μm pore size membrane to generate pre-PSO tissue blocks. These tissue blocks were seeded into 96-well plates, buffered with GelMA gel (Engineering for Life, Suzhou, China). In addition, released single cells were collected and cultured in flasks, coating with gelatin (Sigma-Aldrich, Shanghai, China. Catalog #48722) for further patient-specific primary cultured cancer cell production. Both PSOs and patient-derived primary cells were cultured using primary cell culture medium and placed in a 37°C 5% CO2 incubator. The next day, the activated PSOs adhering to the GelMA gel is ready for the NK killing efficiency test, while the deactivated PSOs will be suspended and washed away.

### Statistical analysis

All data were statistically analyzed using GraphPad Prism8.0 software and IBM SPSS Statistics 25 software. The Kaplan-Meier method was used to calculate survival rates, with the log-rank test used to determine significance. A Cox proportional hazards regression model was used to identify possible independent prognostic factors for the overall survival. Chi-square test was used to analyze data for categorical variables. One-way ANOVA and Newman-Keuls were used for pairwise comparisons between multiple sets of data. Two-way ANOVA and Sidak *t* test were used to analyze the difference with tumor size in different group of mice and Tukey test were used to analyze the results of non-invasive cell analyzer. *P* values < 0.05 were considered statistically significant.

## Results

**Figure 1:**
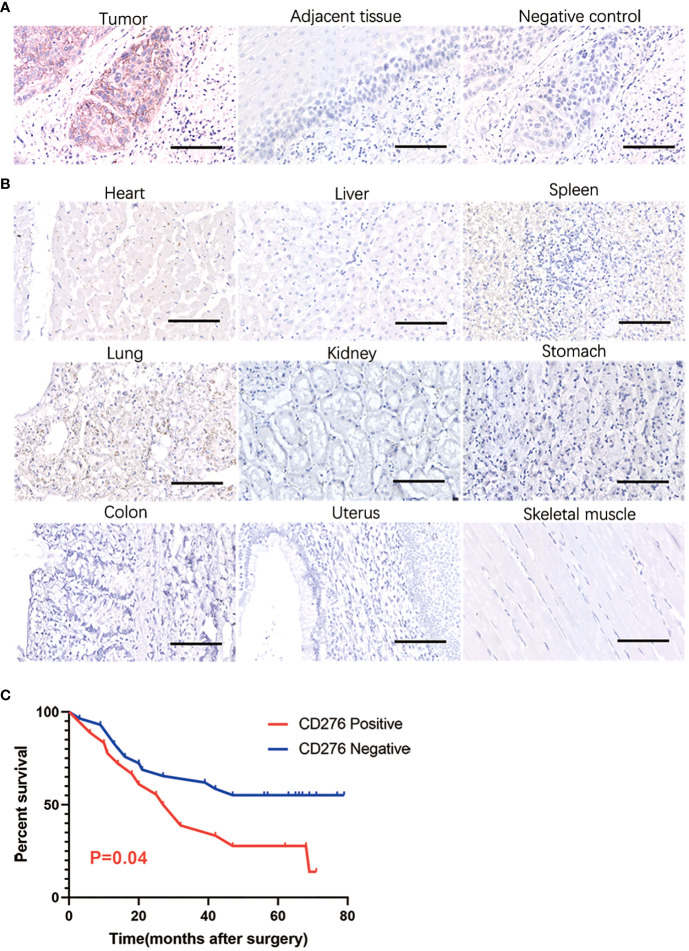
IHC showing CD276 specifically expressing in human esophageal squamous cell carcinoma. In our immunohistochemistry staining of CD276 on normal control samples (adjacent esophageal epithelium located more than 2cm away from the proximal end of esophageal squamous cell carcinoma), we observed no detectable positive CD276 staining in the normal esophageal epithelium. Similarly, in our investigations, we did not observe specific CD276 staining in cell types within human esophageal squamous cell carcinomas **(A)**, as well as in various human tissues, including heart, liver, spleen, lung, kidney, stomach, colon, uterus, and skeletal muscle, as determined from autopsy specimens **(B)**. Among stage III patients, those with CD276-positive ESCCs had significantly worse overall survival than those with CD276-negative ESCCs (P=0.04) **(C)**. Scale bars: 100 µm.

CD276 staining was specifically detected on the ESCC membrane in 51.43% (54/105) of all stages of patients and in 51.35% (38/74) of stages III and IV. In our immunohistochemistry staining of CD276 on normal control samples (adjacent esophageal epithelium located more than 2cm away from the proximal end of esophageal squamous cell carcinoma), we observed no detectable positive CD276 staining in the normal esophageal epithelium. Similarly, in our investigations, we did not observe specific CD276 staining in cell types within human esophageal squamous cell carcinomas (**Figure 1A**), as well as in various human tissues, including heart, liver, spleen, lung, kidney, stomach, colon, uterus, and skeletal muscle, as determined from autopsy specimens (**Figure 1B**). However, it is important to note that the Human Protein Atlas (https://www.proteinatlas.org/ENSG00000103855-CD276/pathology) suggests the presence of CD276 in several human tissues, such as lung, colon, endometrium, etc., at a “medium” rate. This inconsistency in CD276 expression may arise due to interindividual variation in CD276 expression levels and the utilization of diverse CD276 detection techniques. Among stage III patients, those with CD276-positive ESCCs had significantly worse overall survival than those with CD276-negative ESCCs (P=0.04) (**Figure 1C**). **Table 1** provides an overview of patients’ clinical features and their CD276 expression. Cox regression analysis was conducted on various potential prognostic factors (age, gender, depth of primary cancer invasion, lymph node metastasis, TNM stages, CD276 expression, tumor site, and differentiation) for overall survival. The results showed that, in ESCC patients following surgery, lymph node metastasis (P=0.018) rather than CD276 expression (P=0.128) was an independent prognostic factor, as demonstrated in **Supplementary Table 1**.

**Figure 2:**
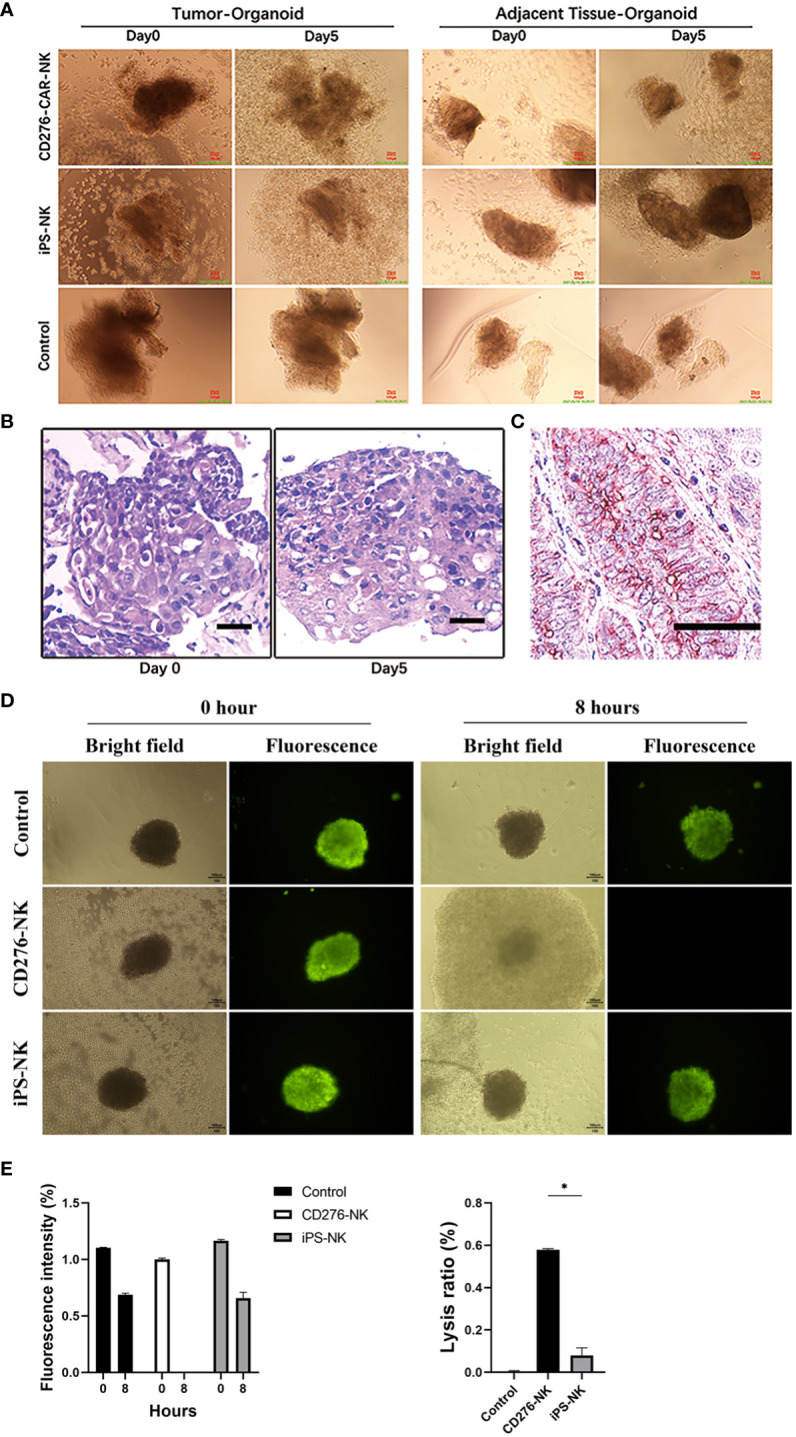
The CD276-expressing ESCC PSO specifically disintegrated by iPSC CD276-targeted CAR-NK cells. The ESCC PSO (primary cancers surgically resected) and NC PSO (adjacent esophageal epithelium located 2cm away from the proximal edge of ESCC) were obtained and processed from the surgical specimens. When co-cultured with iPSC-derived CD276-targeted CAR-NK cells at the same dosage from day 1 to day 5 **(A)**, the CD276-expressing ESCC PSO experienced significant disintegration and suspension, while the CD276-negative NC PSO persisted and continued to grow during the same period. Moreover, the CD276-expressing ESCC PSO maintained its viability and growth morphology when cultured with iPSC NK cells or NK-culturing medium until day 5. These observations indicate that the characteristics of the PSO remain unaffected, except when lysed by CAR-NK cells, when evaluating the efficacy of NK or CAR-NK cells. In **(B)**, the viable normal organoids were embedded in paraffin and sectioned with a thickness of 4um. Hematoxylin and eosin (HE) staining was subsequently performed on the organoid sections. The tissue architecture and cellular morphology of the organoids appeared similar between day 0 and day 5. This implies that the organoids remained viable and exhibited normal growth during the early stage. Scale bar, 30 µm. **(C)** was the immunohistochemistry staining of CD276 in a representative ESCC patient sample section (176114). Scale bar, 50 µm. **(D)** showed the fluorescence of cancer cell vitality in PSO cultured with CD276-CAR NK cells, iPSC-NK cells and NK-culturing medium respectively at 0 and 8 hours. In **(E)**, relative fluorescence intensity of cancer cell vitality in PSO faded away at 8 hours when co-cultured with CD276-CAR NK cells, but remained high vitality in any of the other groups at the same time (left). The PSO lysis ratio in the CD276-CAR NK group was significantly higher than any of the other groups (right). *P < 0.05.

The ESCC PSO and the NC PSO were procured from surgically resected primary cancers and adjacent esophageal epithelium 2cm away from the near edge of ESCC. When co-cultured with iPSC CD276-targeted CAR-NK cells at the same dose from day 1 to day 5 (**Figure 2A**), the CD276-expressing ESCC PSO significantly disintegrated and suspended, while the CD276-negative NC PSO persisted and grew at the same time. Furthermore, while the CD276-expressing ESCC PSO maintained its living and growing morphology when cultured with iPSC NK cells or NK-culturing medium until day 5, it disintegrated when co-cultured with the iPSC CD276-targeted CAR-NK cells until day 5. A 12-second video created from consecutive photos of the experimental group over 41.5 hours showed that an increasing number of iPSC CD276-targeted CAR-NK cells moved forward, assembled closely around, and infiltrated the ESCC PSO. The ESCC PSO gradually became shallower and eventually disintegrated (**Supplementary Video 1**). Histologically, the tissue architecture and cell morphology of the early stages of the surviving PSO were similar to those of the parent ESCC (**Figure 2B**). Immunohistochemistry confirmed that CD276 staining was predominantly located on the membrane of the parent ESCC (**Figure 2C**). The fluorescence of cell vitality in ESCC PSO faded away at 8 hours when co-cultured with iPSC CD276-targeted CAR-NK cells, but remained high vitality when cultured with iPSC NK cells or NK-culturing medium at the same time (**Figure 2D**). The PSO lysis ratio in the iPSC CD276-targeted CAR-NK group was significantly higher than any of the other groups (**Figure 2E**).

**Figure 3:**
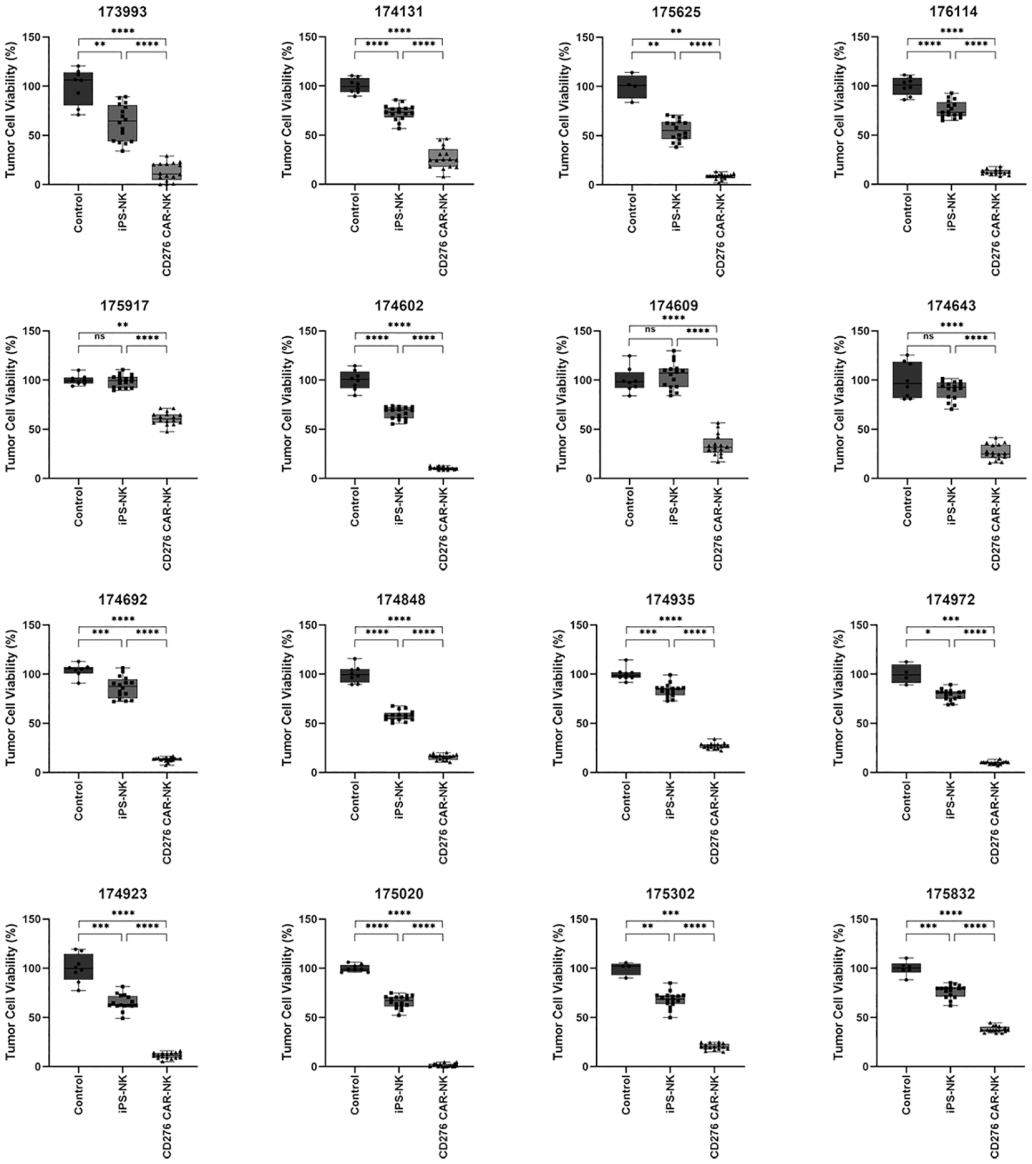
The significant cytotoxicity of iPSC CD276-targeted CAR-NK cells against CD276-expressing primary cultured ESCC cells detected by using CCK-8 assay. We measured the viability of primary cultured ESCC cells from a total of 16 CD276-positive ESCC patients. These cells were co-cultured with iPSC CD276-targeted CAR-NK cells (with replication of 16 times in 16 wells for each patient) and compared with co-cultures involving iPSC NK cells (replicated 16 times in 16 wells for each patient) and the NK cell culture medium (replicated 8 times in 8 wells for each patient) groups. The iPSC CD276-targeted CAR-NK cells displayed significant cytotoxicity against CD276-expressing primary cultured ESCC cells in comparison to the iPSC NK cell group or the NK cell culture medium blank control (refer to **Figure 3**). All the ratios of NK: ESCC cells were 1:2. *P < 0.05, **P < 0.01, ***P < 0.001, ****P < 0.0001, One-way ANOVA and Newman-Keuls test, ns: non significance.

Following ESCC PSO manufacture, the released single cells were collected and cultured in flasks to generate patient-specific primary cultured cancer cells. To demonstrate the significant cytotoxicity of iPSC CD276-targeted CAR-NK cells against CD276-expressing primary cultured ESCC cells, we conducted a CCK-8 assay. We measured the viability of primary cultured ESCC cells from a total of 16 cases of CD276-positive ESCC patients (**Supplementary Figure 1**). These cells were co-cultured with iPSC CD276-targeted CAR-NK cells (with replication of 16 times in 16 wells for each patient) and compared with co-cultures involving iPSC NK cells (replicated 16 times in 16 wells for each patient) and the NK cell culture medium (replicated 8 times in 8 wells for each patient) groups. The results of this analysis indicated that iPSC CD276-targeted CAR-NK cells displayed significant cytotoxicity against CD276-expressing primary cultured ESCC cells in comparison to the iPSC NK cell group or the NK cell culture medium blank control (**Figure 3**).

**Figure 4:**
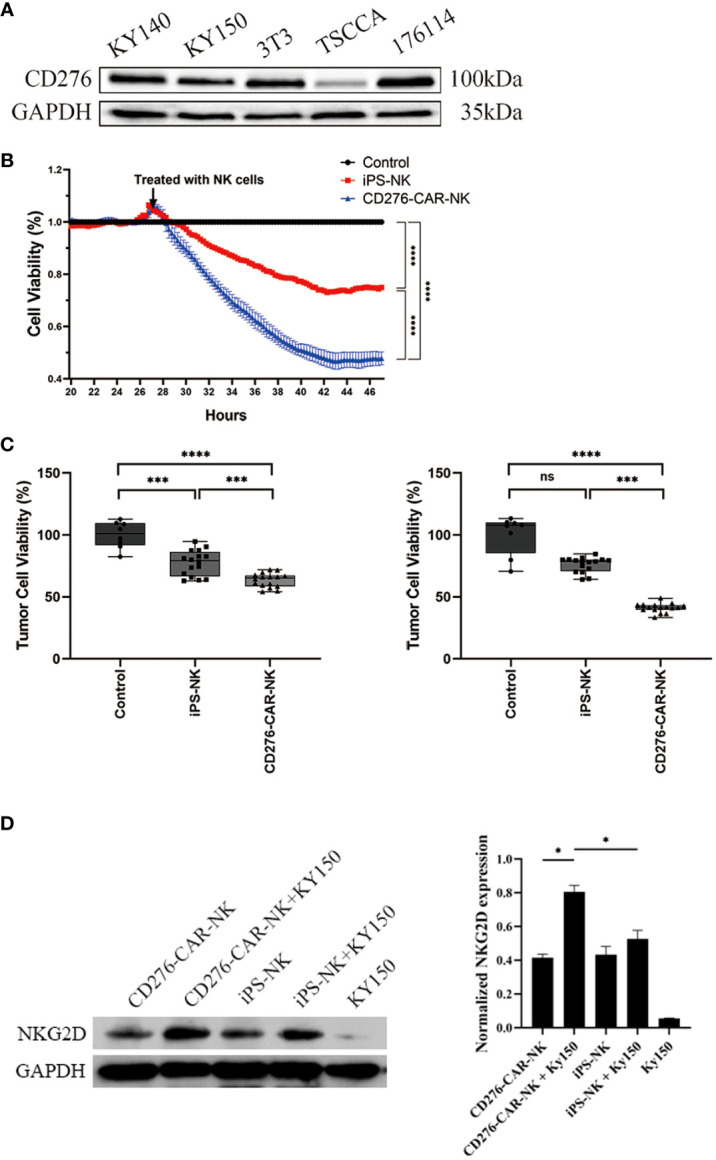
The significant cytotoxicity of iPSC CD276-targeted CAR-NK cells against CD276-expressing ESCC cell line. **(A)** Western blot was conducted to detect the CD276 expression in kyse150 cell line, with specific positive band at 100kd. GADPH served as a loading control. **(B)** Using the xCELLigence RTCA, we found that the viability of Kyse-150 cells co-cultured with iPSC CD276-targeted CAR-NKs declined gradually and more rapidly than those co-cultured with iPSC NK cells (all P < 0.0001) or the NK-culturing medium blank control (all P < 0.0001). The ratio of NK: Kyse-150 corresponds to 1:2. ****P < 0.0001 Two-way ANOVA and Tukey test. **(C)** The CCK8 assay validated that the viability of Kyse-150 cells co-cultured with iPSC CD276-targeted CAR-NKs significantly decreased (with the ratio of the number of effector cells to target cells = 1:4 and 1:2, respectively), compared with either the iPSC NK cells group (all P < 0.001) or the NK-culturing medium group (all P < 0.0001). ***P < 0.001, ****P < 0.0001, One-way ANOVA and Newman-Keuls test. **(D)** illustrates the expression of NKG2D on NK cells and its increase upon exposure to target KY150 cells. NKG2D expression was calibrated using GAPDH as a loading control. The NKG2D expression significantly increased when CD276-positive kyse150 cells co-cultured with the CD276-targeted CAR-NKs, compared with those co-cultured with iPSC NKs, and compared with the iPSC CD276-targeted CAR-NKs, iPSC NKs and kyse150 cells only respectively (right). *P < 0.05, ns: non significance.

To test the efficacy of iPSC CD276-targeted CAR-NK cells against CD276-expressing ESCC cell lines (Kyse-150), we also utilized the traditional preclinical *in vitro* model. We conducted western blotting to detect CD276 expression in the Kyse150 cell line, which produced a specific positive band at 100kd (**Figure 4A**). The viability of Kyse-150 cells was measured in a time and dose-dependent manner when co-cultured with iPSC CD276-targeted CAR-NK cells, iPSC NK cells, and the NK-culturing medium blank control.

Using the xCELLigence RTCA, we found that the viability of Kyse-150 cells co-cultured with iPSC CD276-targeted CAR-NKs declined gradually and more rapidly than those co-cultured with iPSC NK cells (all P < 0.0001) or the NK-culturing medium blank control (all P < 0.0001) (**Figure 4B**). The CCK8 assay validated that the viability of Kyse-150 cells co-cultured with iPSC CD276-targeted CAR-NKs significantly decreased (with the ratio of the number of effector cells to target cells = 1:4 and 1:2, respectively), compared with either the iPSC NK cells group (all P < 0.001) or the NK-culturing medium group (all P < 0.0001) (**Figure 4C**). NKG2D, an essential activation receptor of NK cells was detected with Weston blot in each group (**Figure 4D**). The NKG2D expression significantly increased when CD276-positive kyse150 cells co-cultured with the iPSC CD276-targeted CAR-NKs, compared with those co-cultured with iPSC NKs, and compared with the iPSC CD276-targeted CAR-NKs, iPSC NKs and kyse150 cells individually, respectively. GADPH served as a loading control (**Figure 4D**).

**Figure 5:**
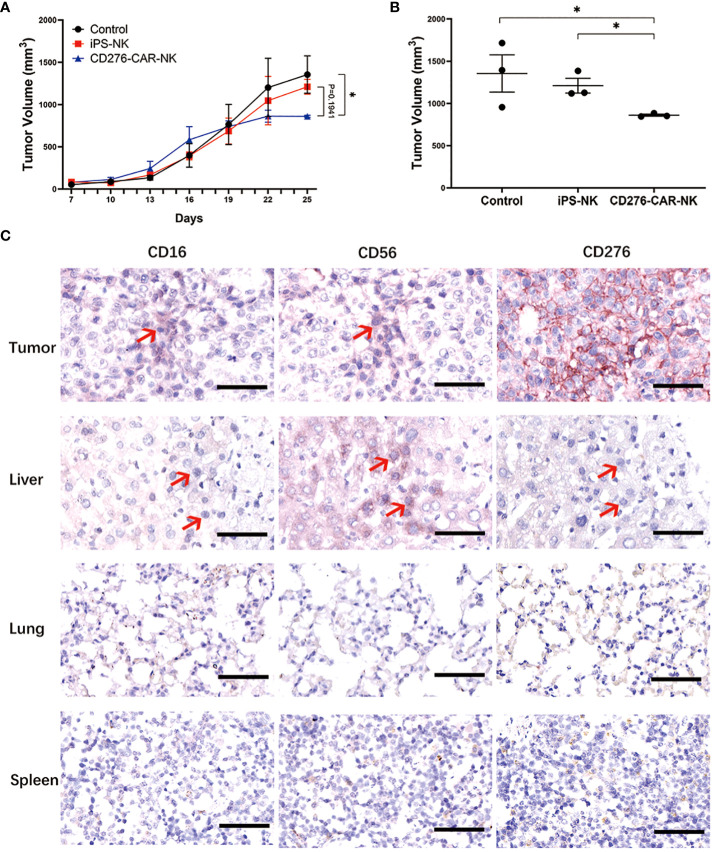
The efficacy of iPSC CD276-targeted CAR-NK cells against CD276-expressing ESCC cell line BNDG mice xenograft. **(A)** Presents a comparison of tumor growth curves among the three groups over the initial 25-day period. We observed a significant deceleration in xenograft tumor growth in the iPSC CD276-targeted CAR-NK group compared to the blank control group (P<0.05) **(A)**. However, the difference in tumor growth between the iPSC CD276-targeted CAR-NK group and the iPSC NK cell group did not reach statistical significance (P=0.1941) when considering the overall comparison of the two curves throughout the entire 25-day observation period **(A)**. The difference of each group (n = 3/group) was analyzed by two-way ANOVA and Sidak t test. *P < 0.05. **(B)** displays the differences in tumor volume among the three groups on day 25. At day 25, the tumor volume in the iPSC CD276-targeted CAR-NK group was significantly smaller than both the iPSC NK cell group (P<0.05) and the blank control group **(B)**. *P < 0.05. **(C)** Immunohistochemical staining of CD16, CD56 and CD276 in tumor, liver, lung and spleen of one ESCC xenograft mouse in CD276 CAR NK group. Scale bar, 50 µm. Red arrows point to human CD276-targeted CAR NK cells in mouse tissues.

To evaluate the efficacy of iPSC CD276-targeted CAR-NK cells against ESCC cells, we generated a CD276-expressing ESCC cell line (Kyse-150) xenograft BNDG mouse model. In total, 9 ESCC cell xenograft BNDG mice were utilized and divided into three groups based on body weight and tumor size. IPSC CD276-targeted CAR-NK cells, iPSC NK cells, and a blank control injection were administered once a week for three consecutive weeks. **Figure 5A** presents a comparison of tumor growth curves among the three groups over the initial 25-day period. We observed a significant deceleration in xenograft tumor growth in the iPSC CD276-targeted CAR-NK group compared to the blank control group (P<0.05) (**Figure 5A**). However, the difference in tumor growth between the iPSC CD276-targeted CAR-NK group and the iPSC NK cell group did not reach statistical significance (P=0.1941) when considering the overall comparison of the two curves throughout the entire 25-day observation period (**Figure 5A**). **Figure 5B** displays the differences in tumor volume among the three groups on day 25. At day 25, the tumor volume in the iPSC CD276-targeted CAR-NK group was significantly smaller than both the iPSC NK cell group (P<0.05) and the blank control group (**Figure 5B**). Additionally, it is important to note that no weight loss or NK cell therapy-induced mortality was observed in any of the BNDG mice. In accordance with the Guidelines for Endpoints in Animal Study Proposals (version 2022), as provided by the Office of Laboratory Animal Welfare (available at https://oacu.oir.nih.gov/system/files/media/file/2022-04/b13_endpoints_guidelines.pdf), it is recommended that the size of a mouse tumor should not exceed 20 mm, and the tumor burden should not surpass 10% of the body weight. Following these essential principles, we were obliged to terminate our animal experiment within a month as the tumor burden approached nearly 10% of the body weight in all groups, as depicted in **Figure 5**.

Based on the results depicted in **Figure 5C**, we conducted immunohistochemical staining of CD56, CD16, and CD276 on tissue sections derived from a representative xenograft mouse model in the iPSC CD276-targeted CAR-NK group. Our findings indicate that CD56 and/or CD16 positive NK cells were frequently observed in CD276-expressing xenograft tumor tissue and also in the liver. These observations suggest that the exogenous CAR-NK cells exhibited migration activities and travelled through the blood circulation from the tail vein of the mouse to various organs, including the liver, remaining active throughout their migration into the xenograft tumor tissue.

## Discussion

Considering the benefits of CAR-NK over CAR-T, the accessibility of CD276-targeted CAR in prior investigations, and the distinctive CD276 expression on the human ESCC membrane, we initially generated iPSC CD276-targeted CAR-NK cells to evaluate their cytotoxicity against CD276-expressing ESCC. Our approach involved employing patient-specific organoid (PSO) models of CD276-positive ESCC PSO and CD276-negative adjacent epithelium PSO (NC PSO), primary culture of ESCC cells models, along with the KYSE-150 *in vitro* and *in vivo* models. The iPSC NK cells and NK-free media were used as CAR and NK-free controls, respectively. The consistent and significant therapeutic efficacy of the iPSC CD276-targeted CAR-NK cells against ESCC was confirmed in preclinical models. These findings underscore their potential as promising therapeutic agents to treat patients with CD276-expressing ESCC or other CD276-expressing solid tumors.

In this study, we employed patient-specific organoid (PSO) models, including the CD276-positive ESCC PSO and the CD276-negative adjacent epithelium PSO (NC PSO). Human organoids are a novel experimental model that bridges the gap between animal models and humans, as the evidence obtained from traditional animal models does not entirely reflect human physiological and pathological changes (38). Patient-derived organoids (PDOs) can maintain cellular diversity, preserve native cell-cell interactions, recapitulate defining histological characteristics, and maintain the transcriptomes and mutation profiles of parent tumors. Accumulating evidence indicates that PDOs can predict responses to conventional therapy, including drugs from clinical trials and chimeric antigen receptor T (CAR-T) cell immunotherapy, making them reliable models for timely clinical options (39, 40). Due to the PDOs’ predictable potential of the therapeutic responses in clinic (41), we developed a novel ESCC patient-specific organoid, which included CD276-positive ESCC PSO and the corresponding CD276-negative NC PSO, as primary testing models. We also used primary culture of ESCC cells models, as well as the KYSE-150 *in vitro* and *in vivo* models, to validate the efficacy of iPSC CD276-targeted CAR-NK cells against CD276-expressing human ESCC.

The viable normal organoids were embedded in paraffin and sectioned with a thickness of 4um. Hematoxylin and eosin (HE) staining was subsequently performed on the organoid sections. As illustrated in **Figure 2B**, the tissue architecture and cellular morphology of the organoids appeared similar between day 0 and day 5. This implies that the organoids remained viable and exhibited normal growth during the early stage. The application of this technology had been previously described in our earlier publication (14). When co-cultured with iPSC-derived CD276-targeted CAR-NK cells at the same dosage from day 1 to day 5 (**Figure 2A**), the CD276-expressing ESCC PSO experienced significant disintegration and suspension, while the CD276-negative NC PSO persisted and continued to grow during the same period. Moreover, the CD276-expressing ESCC PSO maintained its viability and growth morphology when cultured with iPSC NK cells or NK-culturing medium until day 5. These observations indicate that the characteristics of the PSO remain unaffected, except when lysed by CAR-NK cells, when evaluating the efficacy of NK or CAR-NK cells.

Considering the potential for on-target/off-tumor effects, despite the generally lower risk of off-target effects associated with NK-CAR cells, owing to their non-MHC-restricted recognition. As demonstrated in **Figure 1A**, ESCC PSO (primary cancers surgically resected) and NC PSO (adjacent esophageal epithelium 2cm away from the proximal edge of ESCC) were co-cultured with iPSC CD276-targeted CAR-NK cells at the same dosage from day 1 to day 5 (**Figure 2A**). Notably, the CD276-expressing ESCC PSO exhibited significant disintegration and suspension, while the CD276-negative NC PSO persisted and continued to grow during the same period. Furthermore, while the CD276-expressing ESCC PSO maintained its viable and growing morphology when cultured with iPSC NK cells or NK-culturing medium until day 5, it disintegrated when co-cultured with iPSC CD276-targeted CAR-NK cells until day 5. In a 12-second video composed of consecutive photos captured over 41.5 hours, an increasing number of iPSC CD276-targeted CAR-NK cells were observed to migrate towards, closely assemble around, and infiltrate the ESCC PSO. Consequently, the ESCC PSO gradually became shallower and eventually disintegrated (**Supplementary Video 1**). The differential response of CD276-positive ESCC PSO and CD276-negative NC PSO following iPSC CD276-targeted CAR-NK treatment indicates the on-target effect and the potential off-tumor toxicity of iPSC CD276-targeted CAR-NK in future clinical trials. CAR-NK cell therapy has the potential to enhance on-target effects compared to NK cells, while also avoiding cytokine release syndrome (CRS) associated with the cytotoxicity effect of CAR-T cells.

The unique advantages of CAR-NK cells in comparison to CAR-T-based therapy are numerous. NK cells possess broader immune activating pathways and cytotoxicity mechanisms, can be activated through autologous and allogeneic effector functions independent of MHC-presentation, and exhibit innate activity without requiring antigen priming (9, 42, 43). This makes CAR-NK cells an incredibly attractive prospect for the treatment of a wide range of conditions. Recently, hematopoietic stem cell (HSC) and iPSC cell-derived natural killer cells have emerged as a promising platform for emerging therapeutic functions. The ideal therapeutic CAR-expressing identity, reproducible differentiation protocols, and scalable cellular populations, while maintaining identity, are exempt from patient-to-patient variation and cellular exhaustion or senescence. Additionally, these cell sources are open to various layers of manufacturing quality controls, and compatible with off-the-shelf production (44–46). The efficacy of iPSC CD276-targeted CAR-NK cells against CD276-expressing human ESCC was validated in preclinical testing models, demonstrating the feasibility of induced pluripotent stem cell-derived CAR-targeted natural killer cells.

In our study, we found that over half of the ESCC in advanced stages exhibit CD276 expression, with positive staining primarily detected on the ESCC cell membrane. Notably, there was no positive CD276 staining detected in tissue sections from human heart, liver, spleen, lung, kidney, stomach, colon, uterus or skeletal muscle. CD276-positive ESCCs in stage III exhibited significantly unfavorable overall survival compared to CD276-negative ESCCs. However, our analysis using Cox regression showed that lymph node metastasis, rather than CD276 expression, was an independent prognostic predictor for ESCC patients after surgery. While CD276 is commonly overexpressed in ESCC, it has also been reported in a variety of other solid tumors such as head and neck squamous cell carcinoma, lung cancer, prostate cancer, and breast cancer. This identification of CD276 as a cancer-associated cell surface antigen in both ESCC and other solid tumors suggests that it could function as a useful CAR targeting marker for engineering NK therapy.

It is important to recognize that the success of investigational products in traditional preclinical models such as cancer cell lines *in vitro* and *in vivo* mouse xenograft models does not guarantee success in clinical trials as they often prove invalid (47, 48). Conversely, an investigational product that fails to exhibit significant efficacy in traditional preclinical models may still be clinically effective, especially if corresponding patient-derived organoid (PDO) models demonstrate efficacy. As a result, the inability of preclinical models to predict efficacy in upcoming clinical trials has become a major issue in investigational product clinical translational research. Our study’s use of novel ESCC patient-specific organoid models, both CD276-positive ESCC PSO and the CD276-negative adjacent epithelium PSO (NC PSO), combined with the corresponding primary culture of ESCC cells, may offer a more accurate preclinical method to test the efficacy of CAR-NK against human solid malignancies.

## Data availability statement

The original contributions presented in the study are included in the article/**Supplementary Material**. Further inquiries can be directed to the corresponding authors.

## Ethics statement

The study was approved by the Ethical Committee of the Cancer Hospital of Shantou University Medical College (No. 2022103) and the informed consent was obtained from all participants. All animal experiments have been approved by the Medical Animal Care & Welfare Committee of Shantou University Medical College (No. SUMC2022-293). The studies were conducted in accordance with the local legislation and institutional requirements.

## Author contributions

XL: Formal analysis, Writing – original draft, Data curation, Investigation, Software. TG: Data curation, Formal analysis, Investigation, Software, Methodology, Project administration, Validation, Visualization, Writing – review & editing. YX: Data curation, Formal analysis, Investigation, Software, Writing – original draft. YLi: Data curation, Investigation, Methodology, Writing – review & editing. YCL: Data curation, Investigation, Methodology, Writing – review & editing. SC: Investigation, Writing – review & editing, Resources. YPC: Investigation, Resources, Writing – review & editing. XW: Investigation, Resources, Writing – review & editing, Validation. DL: Investigation, Resources, Writing – review & editing. YKC: Investigation, Resources, Writing – review & editing. YLin: Investigation, Resources, Writing – review & editing, Funding acquisition. PS: Investigation, Resources, Validation, Writing – review & editing. CL: Investigation, Resources, Writing – review & editing. JG: Investigation, Resources, Validation, Writing – review & editing. HZ: Conceptualization, Funding acquisition, Methodology, Project administration, Resources, Supervision, Writing – original draft, Writing – review & editing. CM: Funding acquisition, Writing – review & editing, Conceptualization, Formal analysis, Methodology, Project administration, Supervision, Writing – original draft. WY: Methodology, Project administration, Resources, Supervision,Writing – review & editing. JMG: Investigation, Validation, Writing – review & editing.

## Supplementary material

Supplementary Figure 1 Immunohistochemistry staining of CD276 on the parent human ESCC tissue sections. The patient derived primary cultured ESCC cells were derived from the 16 cases of CD276-expressing ESCC patients. Scale bar: 100 µm.

Supplementary Figure 2 More fluorescence signals of scFv from CD276-targeted CAR could be detected in CD276 positive Ky150 by Flow cytometer than in CD276 negative cells and in negative controls. The single-chain fragment variable (scFv) of CD276-targeted CAR were generated and linked with his-tag, which could be visualized when combined with fluorescent secondary antibody. Therefore, stained with the his-tag linked scFv from the CD276-targeted CAR, the CD276 positive Ky150 cells had more immunofluorescence detected by flow cytometer, as compared with CD276 negative cells and negative controls. In other words, the results also supported that the iPSC-derived CD276-targeted CAR-NK could recognized and have more affinity with CD276 positive cells rather than CD276 negative ones.

Supplementary Figure 3 The expression of specific markers at different stages from iPSCs to NK-iPSC to CD276-CAR-NK detected by using flow cytometry. In order to validate the CD276-CAR-NK model prior to the preclinical experiment, we performed flow cytometry analysis to examine the phenotype of the cells, starting from iPSCs expressing CD276-CAR, Oct4, SSEA3, SSEA4, TRA-1-60, TRA-1-81 and Nanog. Further differentiation led to the emergence of CD276-CAR-NK progenitors (CD276-CAR+, CD45+, CD34+), finally producing mature CD276-CAR-NK cells (CD276-CAR+, CD45+, CD3-, CD56+, CD16+).

Supplementary Figure 4 The non-relevant CAR-NK (Mock-CAR-NK) cells exhibited similar lysis efficiency to iPSC-NK cells. Using the xCELLigence RTCA, we found that the viability of each of the three CD276 positive cancer cells (EC109, Ky140 or Ky150) co-cultured with iPSC CD276-targeted CAR-NKs declined gradually and more rapidly than those co-cultured with iPSC NK cells, or with Mock-CAR-NK, or with NK-culturing medium blank control. The non-relevant CAR-NK (Mock-CAR-NK) cells exhibited similar lysis efficiency to iPSC-NK cells when co-cultured with each of the three CD276 positive cancer cells (EC109, Ky140 or Ky150).

Supplementary Video 1 iPSC CD276-targeted CAR NK cells infiltrated into and disintegrated the ESCC PSO.

Supplementary Video 2 iPSC CD276-targeted CAR NK cells kill human ESCC line (KYSE150).

Supplementary Video 3 iPSC CD276-targeted CAR NK cells kill patient-specific primary cells of ESCC.
