# Supplementary figures and images for: Efficacy of the induced pluripotent stem cell derived and engineered CD276-targeted CAR-NK cells against human esophageal squamous cell carcinoma
Source: Front Immunol. 2024 Mar 19;15:1337489. doi: 10.3389/fimmu.2024.1337489 (PMC10985341; doi:10.3389/fimmu.2024.1337489)

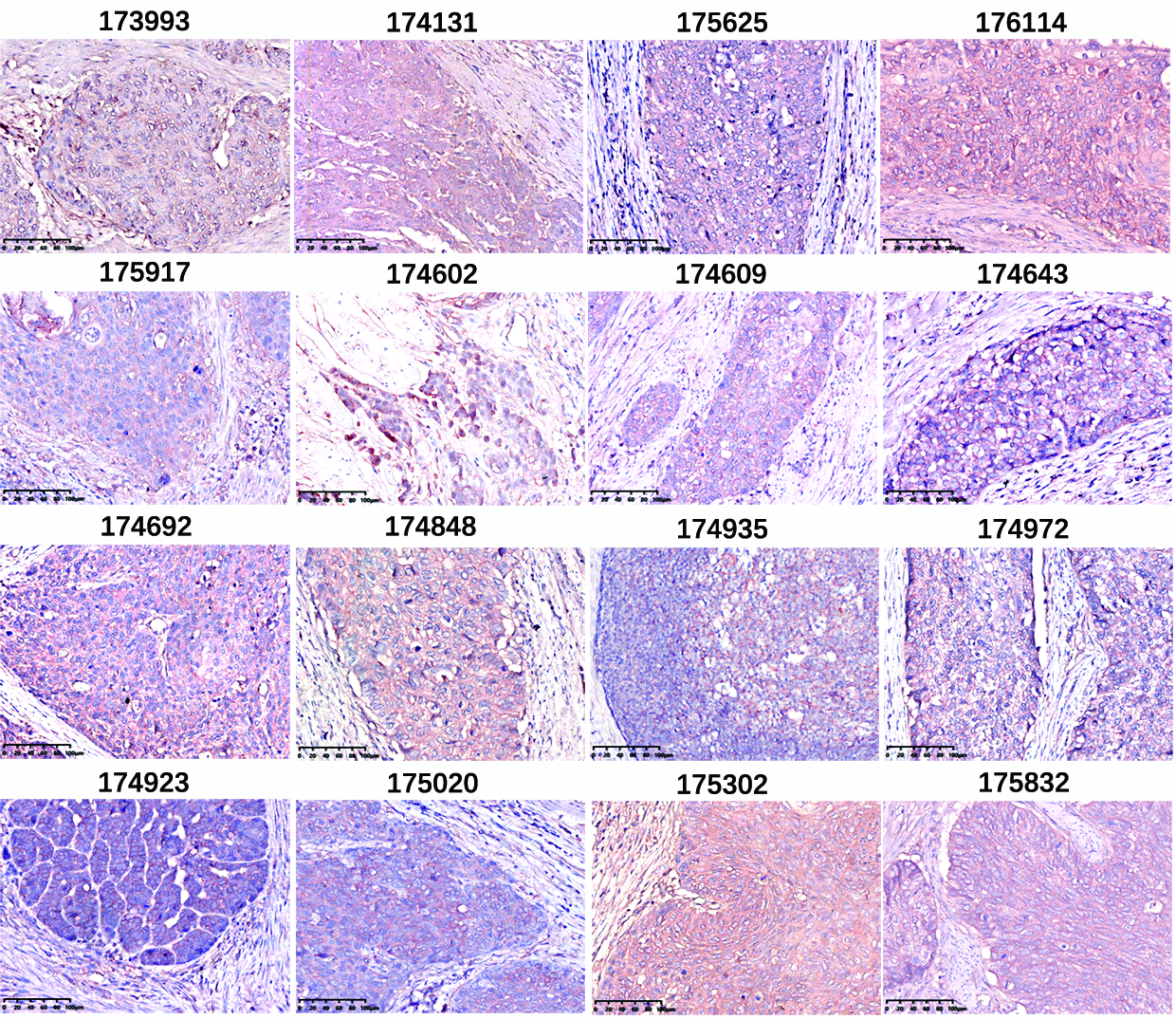

Supplement: Supplementary Figure 1 — Immunohistochemistry staining of CD276 on the parent human ESCC tissue sections. The patient derived primary cultured ESCC cells were derived from the 16 cases of CD276-expressing ESCC patients. Scale bar: 100 µm. [file Image_1.tif]

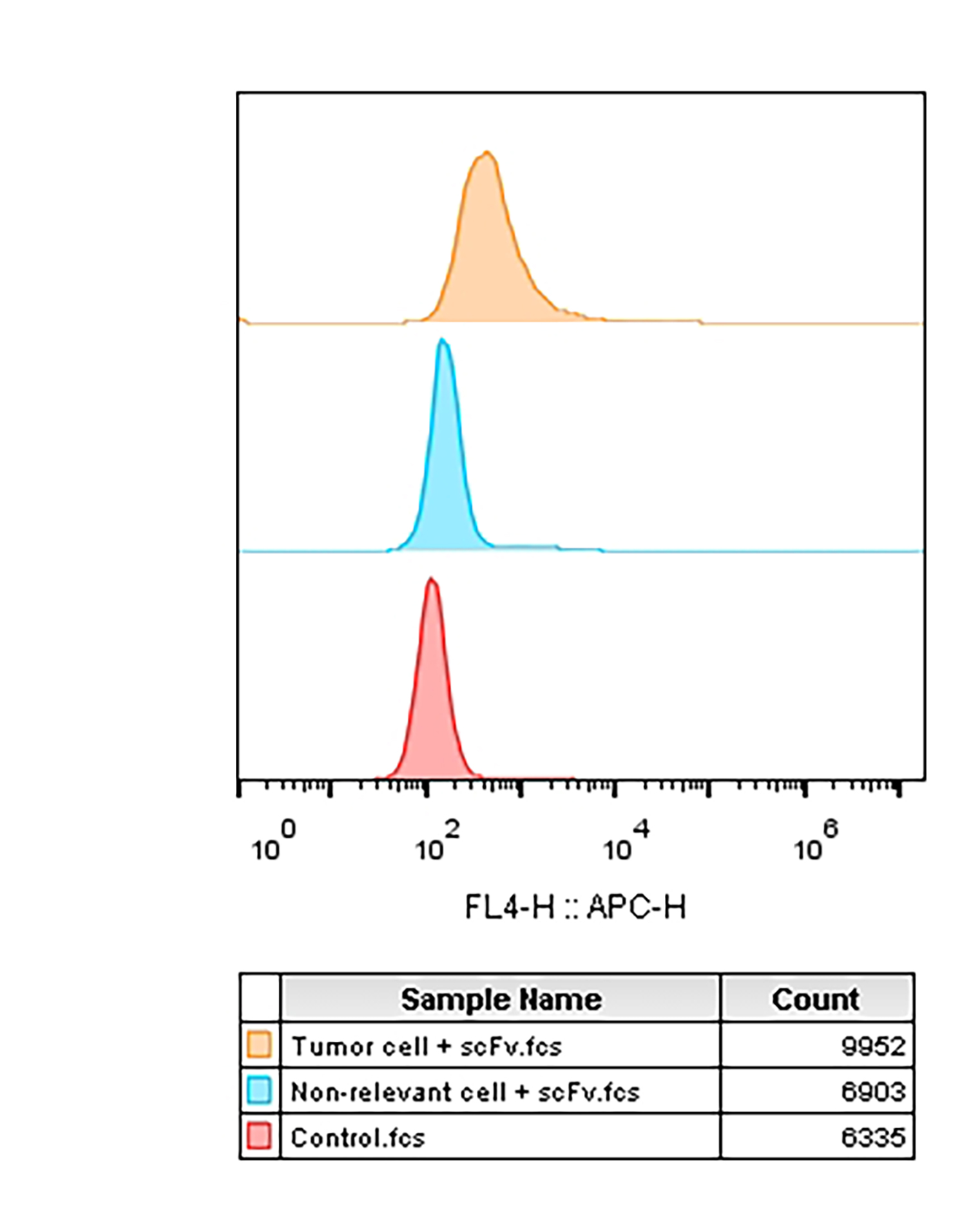

Supplement: Supplementary Figure 2 — More fluorescence signals of scFv from CD276-targeted CAR could be detected in CD276 positive Ky150 by Flow cytometer than in CD276 negative cells and in negative controls. The single-chain fragment variable (scFv) of CD276-targeted CAR were generated and linked with his-tag, which could be visualized when combined with fluorescent secondary antibody. Therefore, stained with the his-tag linked scFv from the CD276-targeted CAR, the CD276 positive Ky150 cells had more immunofluorescence detected by flow cytometer, as compared with CD276 negative cells and negative controls. In other words, the results also supported that the iPSC-derived CD276-targeted CAR-NK could recognized and have more affinity with CD276 positive cells rather than CD276 negative ones. [file Image_2.jpeg]

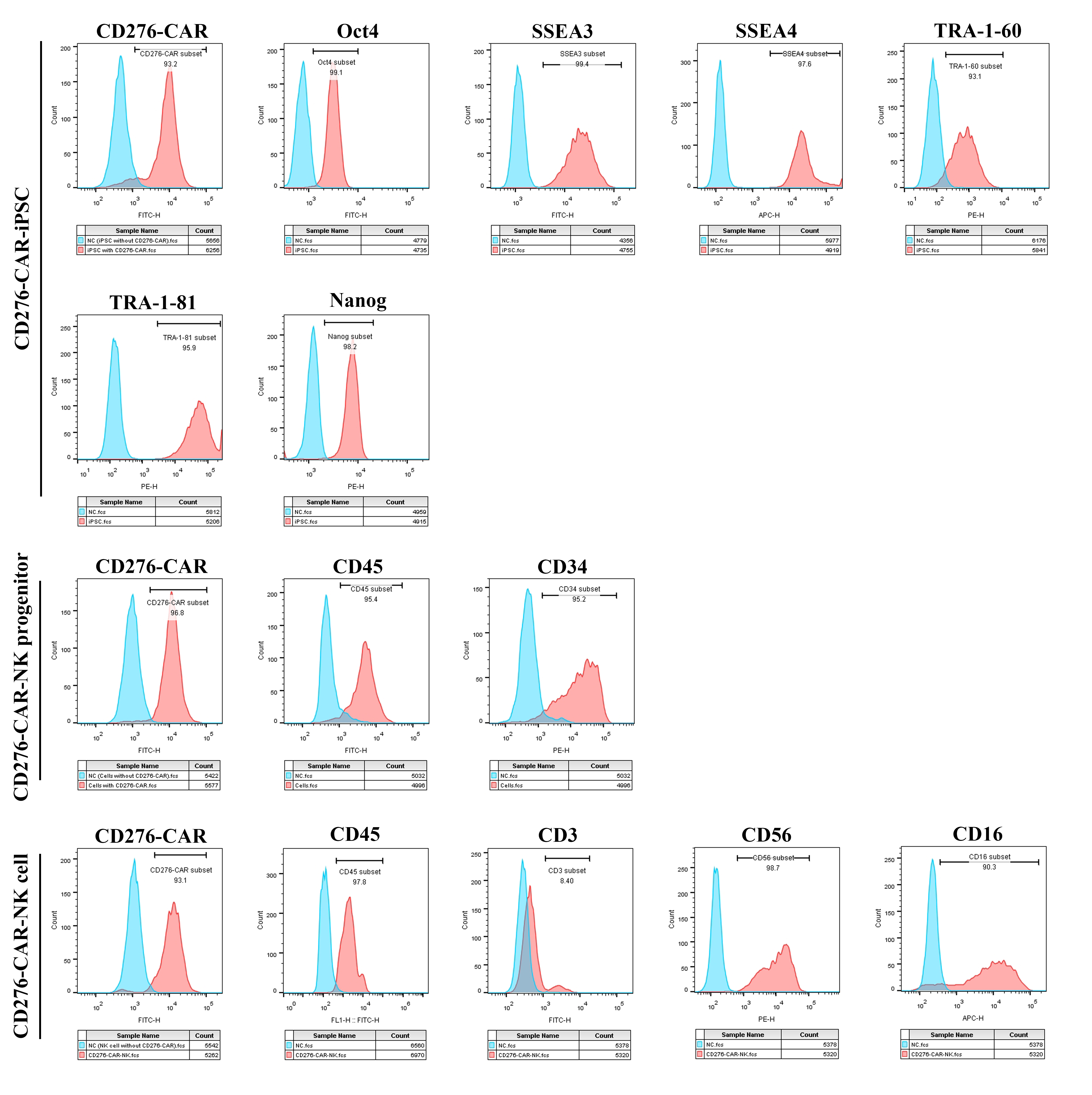

Supplement: Supplementary Figure 3 — The expression of specific markers at different stages from iPSCs to NK-iPSC to CD276-CAR-NK detected by using flow cytometry. In order to validate the CD276-CAR-NK model prior to the preclinical experiment, we performed flow cytometry analysis to examine the phenotype of the cells, starting from iPSCs expressing CD276-CAR, Oct4, SSEA3, SSEA4, TRA-1-60, TRA-1-81 and Nanog. Further differentiation led to the emergence of CD276-CAR-NK progenitors (CD276-CAR+, CD45+, CD34+), finally producing mature CD276-CAR-NK cells (CD276-CAR+, CD45+, CD3-, CD56+, CD16+). [file Image_3.jpeg]

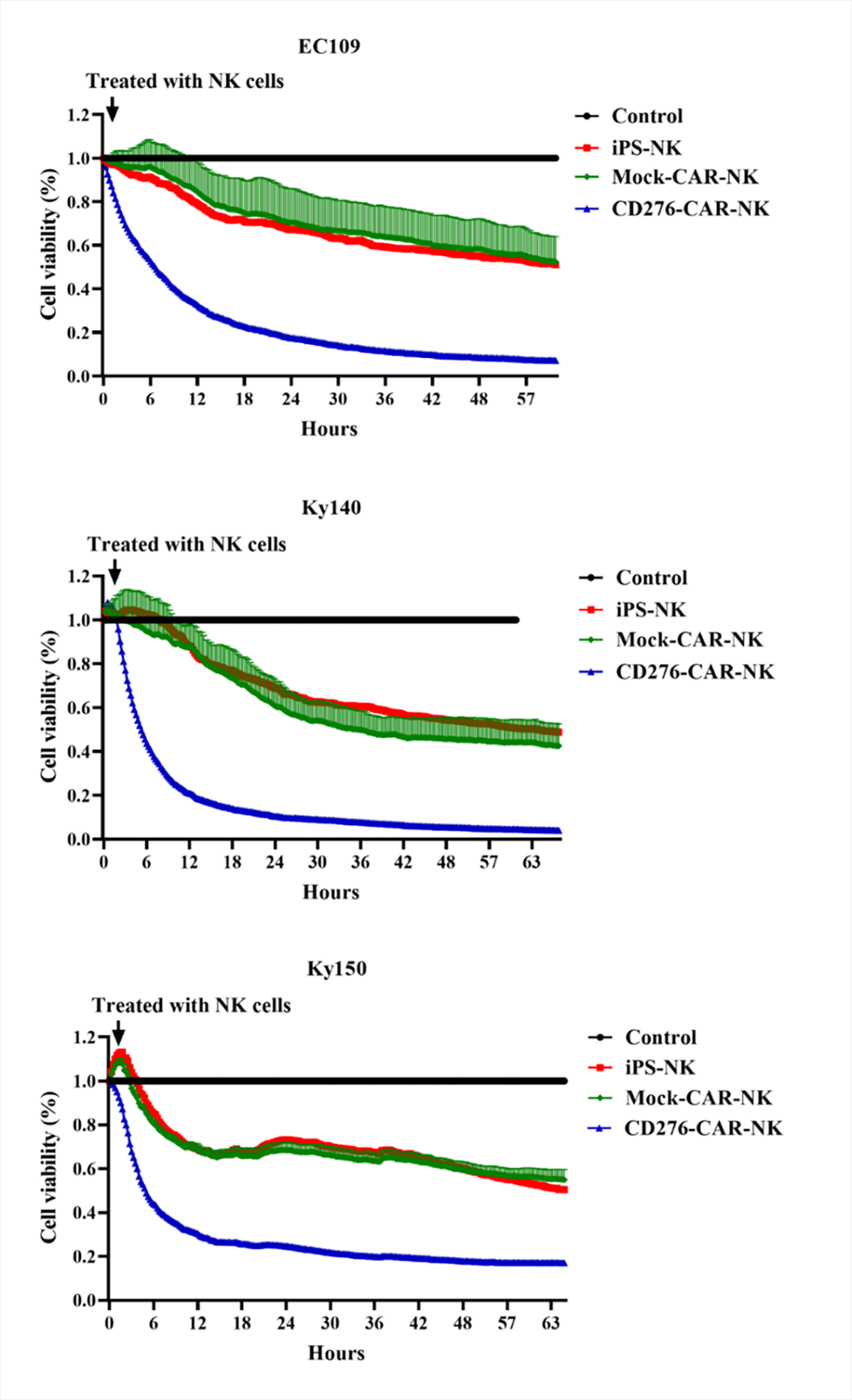

Supplement: Supplementary Figure 4 — The non-relevant CAR-NK (Mock-CAR-NK) cells exhibited similar lysis efficiency to iPSC-NK cells. Using the xCELLigence RTCA, we found that the viability of each of the three CD276 positive cancer cells (EC109, Ky140 or Ky150) co-cultured with iPSC CD276-targeted CAR-NKs declined gradually and more rapidly than those co-cultured with iPSC NK cells, or with Mock-CAR-NK, or with NK-culturing medium blank control. The non-relevant CAR-NK (Mock-CAR-NK) cells exhibited similar lysis efficiency to iPSC-NK cells when co-cultured with each of the three CD276 positive cancer cells (EC109, Ky140 or Ky150). [file Image_4.tif]
