# Supplementary material for: Efficacy of the induced pluripotent stem cell derived and engineered CD276-targeted CAR-NK cells against human esophageal squamous cell carcinoma
Source: Front Immunol. 2024 Mar 19;15:1337489. doi: 10.3389/fimmu.2024.1337489 (PMC10985341; doi:10.3389/fimmu.2024.1337489)
Supplement: Supplementary file 8 [file Table_1.docx]

Table S1: Univariate and multivariate analysis of ESCC patients’ prognostic factors.

| Clinical Parameters | Uni-variate | | | Multi-variate | | |
| --- | --- | --- | --- | --- | --- | --- |
|  | P-value | Hazard ratio | 95% CI | P-value | Hazard ratio | 95% CI |
| Gender |  |  |  |  |  |  |
| Male / Female | 0.472 | 1.421 | 0.545-3.702 | 0.637 | 1.308 | 0.428-4.001 |
| Age (years) |  |  |  |  |  |  |
| < 60 / ≥ 60 | 0.847 | 0.933 | 0.461-1.889 | 0.478 | 0.758 | 0.353-1.628 |
| Depth of invasion |  |  |  |  |  |  |
| T_1+2_/T_3+4_ | 0.159 | 0.357 | 0.085-1.496 | 0.331 | 0.383 | 0.055-2.654 |
| Nodal metastasis |  |  |  |  |  |  |
| N_0_/N_1+2+3_ | **0.010** | 0.307 | 0.126-0.752 | **0.018** | 0.133 | 0.025-0.708 |
| TNM stage |  |  |  |  |  |  |
| S_I+II_ / S_III+IV_ | 0.091 | 0.463 | 0.190-1.131 | 0.297 | 2.640 | 0.425-16.390 |
| CD276 IHC |  |  |  |  |  |  |
| Negative/Positive | 0.375 | 0.726 | 0.358-1.473 | 0.128 | 0.528 | 0.231-1.203 |
| Tumor site |  |  |  |  |  |  |
| Upper/middle/lower | 0.477 | 0.741 | 0.324-1.694 | 0.401 | 1.481 | 0.592-3.703 |
| Differentiation |  |  |  |  |  |  |
| G1/G2/G3 | 0.819 | 1.188 | 0.273-5.168 | 0.755 | 1.293 | 0.258-6.477 |

* The results in bold are statistically significant.
